# *De novo* transcriptome profiling unveils the regulation of phenylpropanoid biosynthesis in unripe *Piper nigrum* berries

**DOI:** 10.1186/s12870-022-03878-1

**Published:** 2022-10-26

**Authors:** Sweda Sreekumar, Kattupalli Divya, Nisha Joy, E. V. Soniya

**Affiliations:** 1 Transdisciplinary Biology, Rajiv Gandhi Centre for Biotechnology (RGCB), Thiruvananthapuram, Kerala India; 2 Research Centre, University of Kerala, Thiruvananthapuram, Kerala India; 3 Biology Centre, Czech Academy of Sciences, Institute of Plant Molecular Biology, České Budějovice, Czech Republic; 4 Centre for Gene Regulation & Expression, School of Life Sciences, University of Dundee, Dundee, Scotland

**Keywords:** Black pepper, Fruits, RNA-Seq, Developmental stages, Secondary metabolite

## Abstract

**Background:**

Black pepper (*Piper nigrum* L.) is rich in bioactive compounds that make it an imperative constituent in traditional medicines. Although the unripe fruits have long been used in different Ayurvedic formulations, the mechanism of gene regulation resulting in the production of the bioactive compounds in black pepper is not much investigated. Exploring the regulatory factors favouring the production of bioactive compounds ultimately help to accumulate the medicinally important content of black pepper. The factors that enhance the biosynthesis of these compounds could be potential candidates for metabolic engineering strategies to obtain a high level production of significant biomolecules.

**Results:**

Being a non-model plant, de novo sequencing technology was used to unravel comprehensive information about the genes and transcription factors that are expressed in mature unripe green berries of *P. nigrum* from which commercially available black pepper is prepared. In this study, the key gene regulations involved in the synthesis of bioactive principles in black pepper was brought out with a focus on the highly expressed phenylpropanoid pathway genes. Quantitative real-time PCR analysis of critical genes and transcription factors in the different developmental stages from bud to the mature green berries provides important information useful for choosing the developmental stage that would be best for the production of a particular bioactive compound. Comparison with a previous study has also been included to understand the relative position of the results obtained from this study.

**Conclusions:**

The current study uncovered significant information regarding the gene expression and regulation responsible for the bioactivity of black pepper. The key transcription factors and enzymes analyzed in this study are promising targets for achieving a high level production of significant biomolecules through metabolic engineering.

**Supplementary Information:**

The online version contains supplementary material available at 10.1186/s12870-022-03878-1.

## Background

Black pepper (*Piper nigrum* L.) is called 'Black Gold' or the ‘King of Spices’ and is a commonly used spice all over the world [1, 2]. The particular pungent taste and diverse medicinal properties of the fruits/berries of black pepper make it a treasured spice. Black pepper is prepared by first immersing the unripe green berries in hot water followed by sun-drying for many days [3]. This spice is effective in treating various diseases including cancer [4, 5].

Black pepper has an array of medicinally active compounds which is the reason for its high demand in Ayurveda. The commercially available black pepper is prepared from the mature green unripe berries and is largely used as a favourite spice as well as a health enhancer [6]. The phytochemicals in black pepper help to remove harmful free radicals that mutate DNA and hence help to protect from cancers and diseases [7]. It is also used as a preservative, perfumery and insecticide [2, 8]. Dietary intake of black pepper increases the secretion of bile, trypsin, chymotrypsin, pancreatic lipase and amylase indicating its beneficial effects on the gastrointestinal system [2]. The bioavailability of curcumin, the bioactive anti-cancer compound in turmeric, can be increased by 20-fold when administered along with piperine [9]. The generalised use of black pepper seems to be due to its ability to enhance the effect of various medicinal herbs.

Despite the immense medicinal benefits from the biochemicals in black pepper, the molecular network shaping the biosynthesis of these secondary metabolites is still largely unknown due to the lack of proper sequence information and fragmented data obtained [10]. In-depth analysis of the unknown mechanisms behind the biosynthesis of medicinally and biologically active secondary metabolites in black pepper has not been accomplished to date. Limited information on the regulation of biosynthesis of specialized metabolites is a hindrance in utilizing medicinally valuable herbs for commercial level production using synthetic biology [11, 12]. It is imperative to understand the genes responsible for the accumulation of biologically active secondary metabolites for the betterment of the quality parameters of the fruit [13]. Next Generation Sequencing (NGS) technology has revolutionized molecular biology by providing high-fidelity sequences and wholesome high-throughput information on transcriptomes [14]. High-throughput data helps to identify critical nodes including enzymes and transcription factors involved in the biosynthetic regulation of bioactive compounds.

Although minor experiments have been carried out based on the bioactive compounds present in pepper, high-throughput platforms have not been utilized yet to uncover the depth of the mechanisms and regulations that are involved in their biosynthesis. Only by dissecting these networks, a suitable metabolic engineering strategy can be adopted and applied in the near future to ensure high-level production of such therapeutically significant compounds. The major genes and regulatory agents should be tracked and a comprehensive understanding of the interplay between the key candidates that help in the biosynthesis of these principles should be portrayed to make the best use of this spice crop in the pharmaceutical industry. The present study has utilized high-throughput transcriptomics to obtain a better understanding of the under-studied aspects of the molecular network behind phenylpropanoid biosynthesis in *P. nigrum* fruits.

## Methods

### Selection of developmental stages of *P. nigrum*

**Fig. 1:**
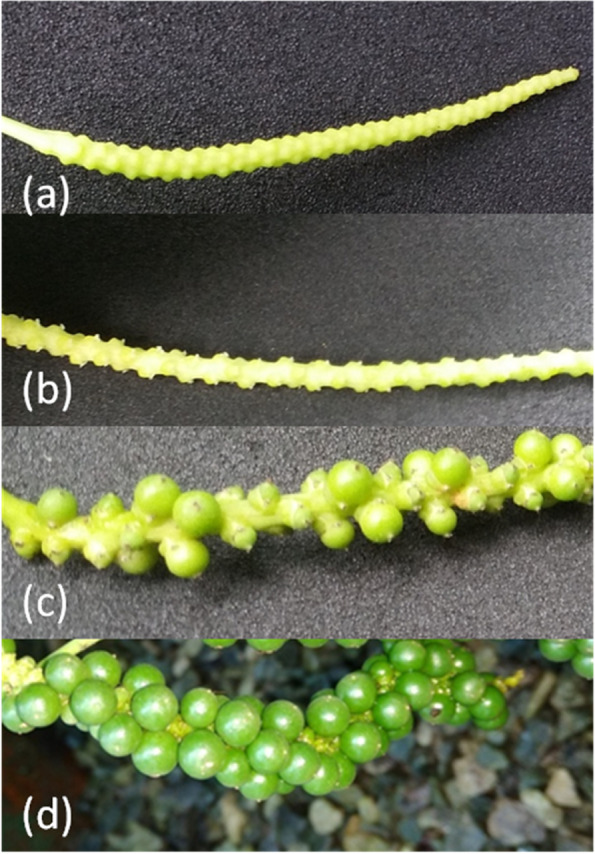
Different developmental stages (**a**) Bud, (**b**) Flower (tiny white), (**c**) Immature young fruit stage 1 (smaller fruits in the spike) and stage 2 (slightly bigger fruits in the spike), (**d**) mature unripe berry

Unripe berries of *P. nigrum *(variety—Panniyur 1) maintained in the green house of Rajiv Gandhi Centre for Biotechnology, Thiruvananthapuram, were used for extracting RNA for transcriptome sequencing.The time of collection of fruits was recorded as days after flowering (DAF). Total RNA was extracted from five different developmental stages and chosen for the quantitative real-time PCR experiments (Fig. 1): Bud, Flower, Immature young fruit [stage 1(30 DAF)], Immature young fruit [stage 2 (60 DAF)], and Unripe berry (120 DAF). The transition from bud stage to fully flowering stage took approximately 12–15 days. The plant samples collected were flash-frozen using liquid nitrogen. They were stored at -80 °C until further use.

### Isolation of RNA

High quality total RNA was extracted from 50-100 mg of tissues from the different developmental stages of *P. nigrum* as previously mentioned, using Spectrum™ Plant Total RNA Kit by standardized procedure (Sigma-Aldrich). RNA isolation was carried out in an RNA-safe environment. Before adding the samples for grinding, pre-cooled the mortar by adding liquid nitrogen for maintaining the sample in a chilled condition. Ground the samples to fine white powder, intermittently adding liquid nitrogen to the mortar. Samples were homogenized with lysis buffer. The lysate was centrifuged to remove debris. Supernatant was mixed with binding buffer and loaded onto Spectrum column and further steps were followed as per manufacturer’s guidelines. RNA was eluted in Nuclease free water (Ambion, USA). The quantity and quality of the RNA was assessed using microvolume spectrophotometer (Colibri, Germany).

### High-throughput sequencing and analysis

*De-novo* transcriptome sequencing of *P. nigrum* unripe fruit was carried out. Workflow for the analysis is given in Additional file 1.The library preparation for RNA-Seq was performed at Genotypic Technology, Bangalore, India, according to Illumina-compatible NEBNext UltraTM Directional RNA Library Prep.

Illumina HiSeq sequencer was used for sequencing 150 bp length paired-end (PE) reads to produce a total of 2, 60, 90, 849 raw sequencing reads. The quality checking was carried out using FastQC (https://www.bioinformatics.babraham.ac.uk/projects/fastqc/). Processing of raw reads was done using Cutadapt tool [15] for adapters and low-quality bases trimming towards 3'-end, with average Phred quality score (Q) of ≥ 30 at each base position. A total of 2, 44, 45, 774 high-quality adapter-free data was used after pre-processing for assembly.

Construction of RNA sequencing libraries was done using Illumina-compatible NEBNext® Ultra™ Directional RNA Library Prep Kit (New England BioLabs, MA, USA).1 μg of total RNA was used for mRNA isolation. This was followed by fragmentation and priming. Further, first strand synthesis was done and then second strand synthesis was carried out. HighPrep magnetic beads (Magbio Genomics Inc, USA) were used for purifying double-stranded cDNA. The resulting cDNA was end-repaired, adenylated and ligated to Illumina multiplex barcode adapters according to the protocol of NEBNext® Ultra™ Directional RNA Library Prep Kit.

Illumina Universal Adapters used:

5’-AATGATACGGCGACCACCGAGATCTACACTCTTTCCCTACACGACGCTCTTCC

GATCT-3’

and Index Adapter:

5’-GATCGGAAGAGCACACGTCTGAACTCCAGTCAC[INDEX]ATCTCGTATGCCGT

CTTCTGCTTG-3’.

Here, [INDEX] is the unique sequence to identify sample-specific sequencing data.

After ligation with the adapter, cDNA was purified with HighPrep beads. Purified cDNA underwent 12 cycles of Indexing-PCR (37 ˚C for 15 min, then denaturation at 98 ˚C for 30 s, followed by cycle of 98 ˚C for 10 s and 65 ˚C for 75 s, then finally 65 ˚C for 5 min) for enriching the adapter-ligated fragments. The PCR product is the sequencing library, which was purified with HighPrep beads. Quality control checking was done. The quantification was done using Qubit fluorometer (ThermoFisher Scientific, MA, USA). The fragment size distribution was analyzed on Agilent 2200 Tapestation.

### Assembly and annotation

Trinitysoftware package [16] was used to assemble the trimmed reads as contigs of the *P. nigrum* transcriptome. Long contig sequences were obtained by continuously joining overlapping reads of particular quality and length. The maximum and minimum length, N50 and average length were determined. CD-HIT open source program [17, 18] was used for clustering the transcripts based on similarity of sequences. The initial analysis included normalizing of read counts for the transcripts to get Reads Per Kilobase of transcript per Million mapped reads (RPKM).

Functional annotation was done using several databases including KAAS (http://www.genome.jp/tools/kaas/), MISA (https://webblast.ipk-gatersleben.de/misa/), GO (http://geneontology.org/) and Uniprot (https://www.uniprot.org/). NCBI-BLAST (https://blast.ncbi.nlm.nih.gov/Blast.cgi) [19] was used for annotation based on sequence homology with protein sequences of ‘Viridiplantae members’ in Uniprot [20]. Cut-off value was set at more than 30% identity and E-value less than 1e-5. Pathway annotation was done by using KAAS server [21] against the reference dataset of Viridiplantae proteins. The reference organisms used were *Arabidopsis thaliana* (ath), *Theobroma cacao* (tcc), *Tarenaya hassleriana* (thj), *Eucalyptus grandis* (egr), *Glycine max* (gmx), *Fragaria vesca* (fve), *Vitis vinifera* (vvi), *Solanum lycopersicum* (sly*), Populus trichocarpa* (pop) and *Oryza sativa japonica* (osa). Simple sequence repeats were identified from assembled transcripts using MISA.

### Expression analysis of selected genes in different developmental stages of fruits

Comparative quantitative real-time Polymerase Chain Reaction (qRT-PCR) experiment was done for selected genes in the different developing stages (bud, flower, young fruit stage 1 (YF1) and stage 2 (YF2), and unripe berries) (Fig. 1) to study the expression of the phenylpropanoid pathway-associated genes and transcription factors observed in the transcriptomic data of *P. nigrum* berries. For this, the total RNA isolated from each developmental stage was used for cDNA synthesis. Forward and reverse primers were designed using IDT (www.idtdna.com) for the selected genes based on the contig sequences obtained from the transcriptomic data (Additional file 2). The self-complementarity of primers was checked using Oligocalc (http://biotools.nubic.northwestern.edu/).

The quantity and quality of the obtained RNA was checked with spectrophotometer (Colibri, Germany). cDNA was synthesized from 1 μg of RNA using a high capacity cDNA reverse transcription kit (Applied Biosystems, Life Technologies). Each reaction mixture containing 2.5 μl SYBR Green Master mix (Applied Biosystems), 1 μl cDNA template (50 ng), and 0.25 ul each of forward and reverse primer (10 ρM) was made up to a total volume of 5 μl with nuclease free water. Real-time experiments were performed in Biosystems 7900 HT Fast Real-Time system with Power SYBR Green qPCR Master Mix (ABI, Life Technologies) in384-well optical reaction plates (Applied Biosystems, USA). The PCR reactions were performed in triplicate under following conditions: 10 min at 95 °C, and 40 cycles of 15 s at 95 °C and 45 s at 60 °C. The cDNA template was replaced with nuclease free water in the case of negative controls. 5.8S ribosomal RNA was used as the reference gene. The analysis was done using the formula 2^−ddCT^ and the standard deviation was represented as error bar [22]. The expression level in bud was arbitrarily set to 1 and the expression in different tissues was compared to that in the bud [23].

We compared the RPKM of selected genes from our data with that of [24] retrieved from NCBI Sequence Read Archive (SRA) (http://www.ncbi.nlm.nih.gov/Traces/sra/; accession number SRS856941) in which they used a mixture of RNA from different stages of fruit (1–10 months post anthesis).

## Results

### NGS library preparation

Sequencing was performed in Illumina HiSeq sequencer with the total RNA extracted from unripe berry sample (Additional file 3).

The sequencing resulted in a total of 2, 60, 90, 849 raw sequencing reads. Total of 24.45 million reads were used for the downstream analysis after pre-processing. On an average of 93.69% of high quality data was retained (Additional file 4).

**Fig. 2:**
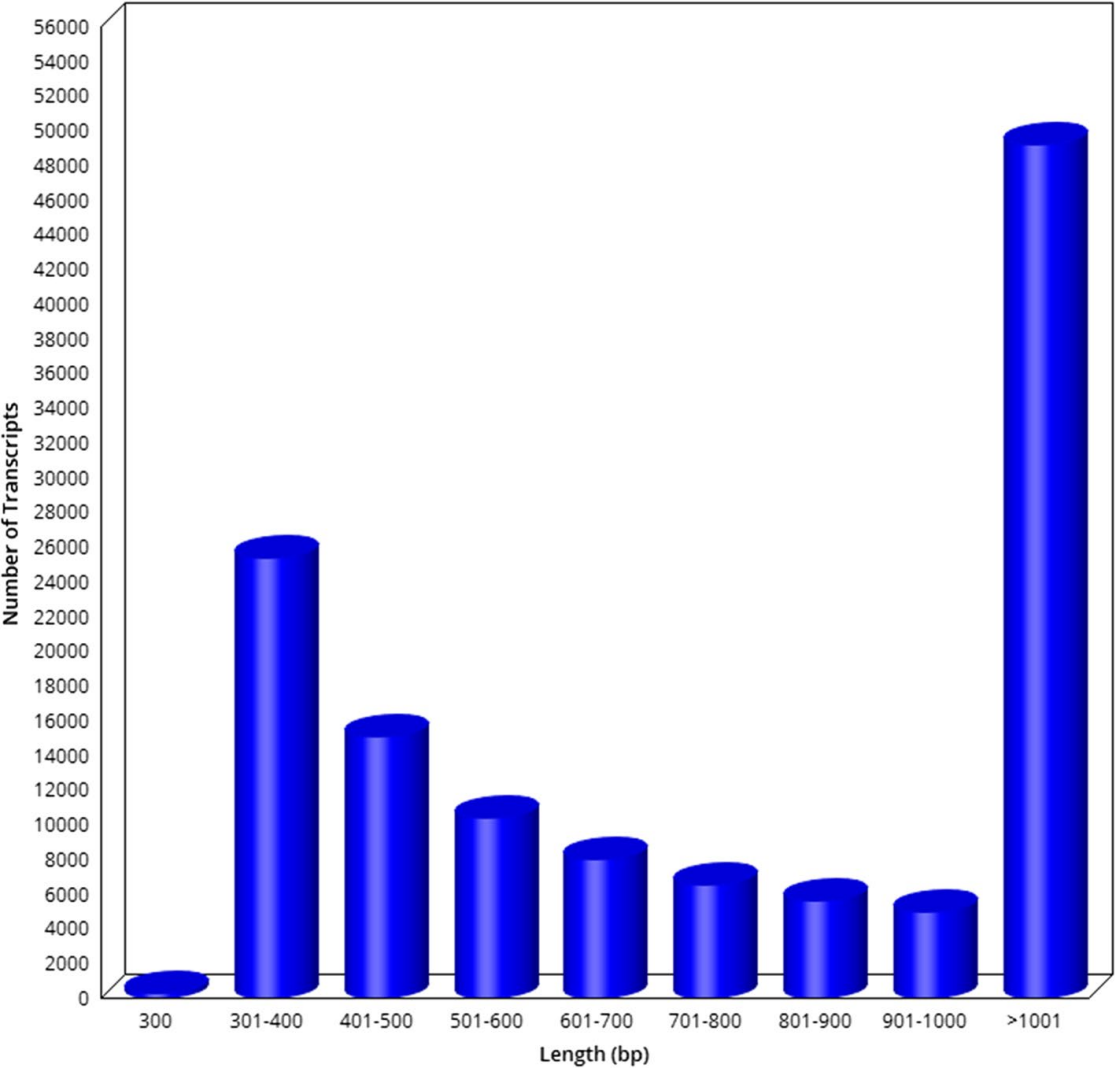
Master unigene length distribution plot

Master transcripts were generated by clustering the transcripts of the sample with 95% similarity. The length distribution of master unigenes is given in Fig. 2.

**Fig. 3:**
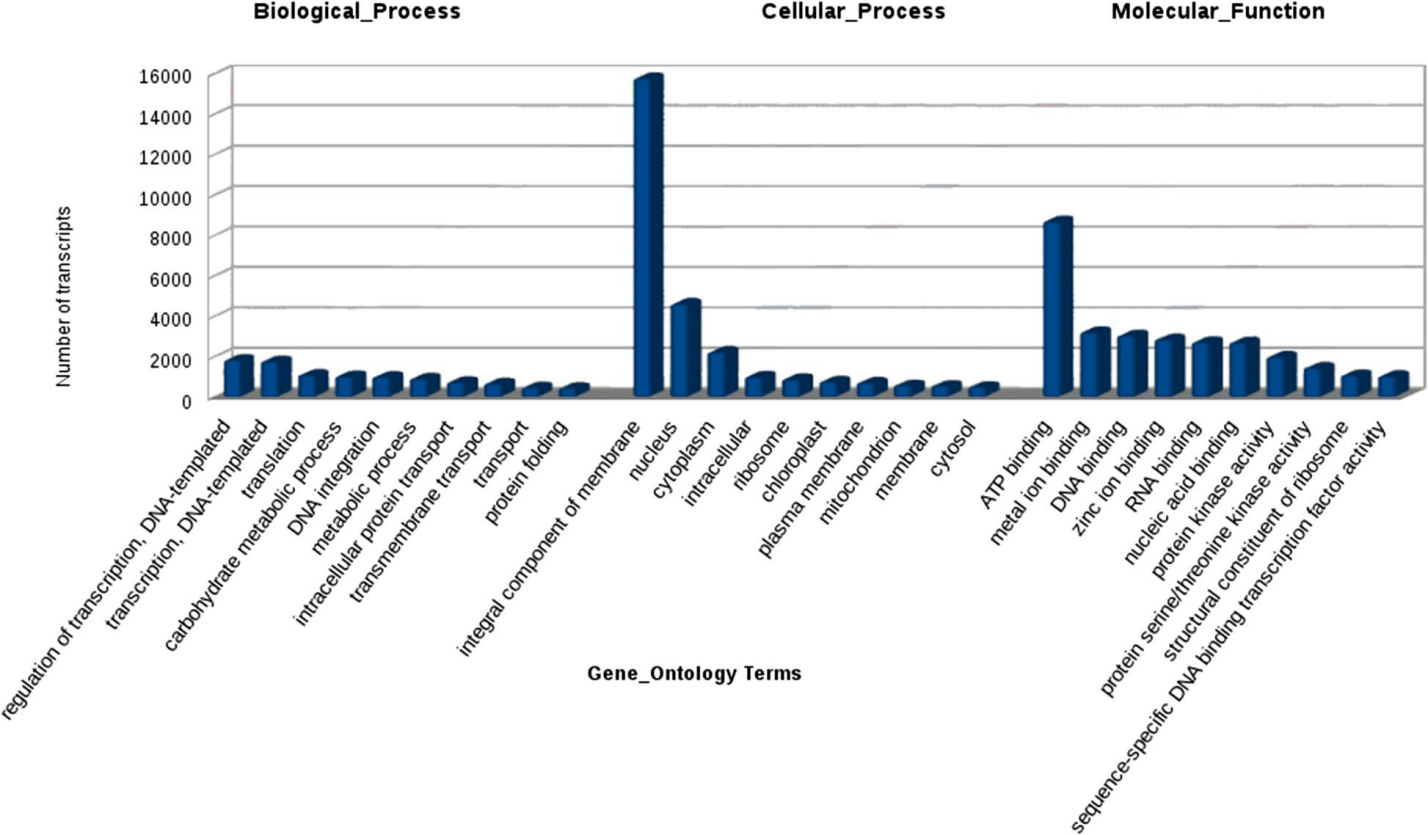
Frequency of most abundant Gene ontology terms

Master transcripts were annotated by the homology search against “Viridiplantae” data from Uniprot containing 51,12,021 protein sequences downloaded on 08/01/2018, using BLAST. Thus 64.50% of the transcripts were functionally annotated. Top 10 abundant Gene Ontology (GO) terms were observed (Fig. 3).

Using Viridiplantae database, 47,085 transcripts with high similarity (E-value < 1e-60) were observed while using BLAST i.e., the possible hits of similar score for these transcripts that could be obtained by mere chance ranged from 1e-60 to 1e-5. This low E-value indicates high significance and were considered for further analysis. The E-value of the rest of the transcripts ranged from 1e-5 to 0 (Additional file 5). Since similarity of sequences is directly proportional to homology, the similarity distribution with reference was analysed. 15,709 of the sequences had a strong similarity of higher than 80% and 64,655 had below 80% similarity (Additional file 6).

**Fig. 4:**
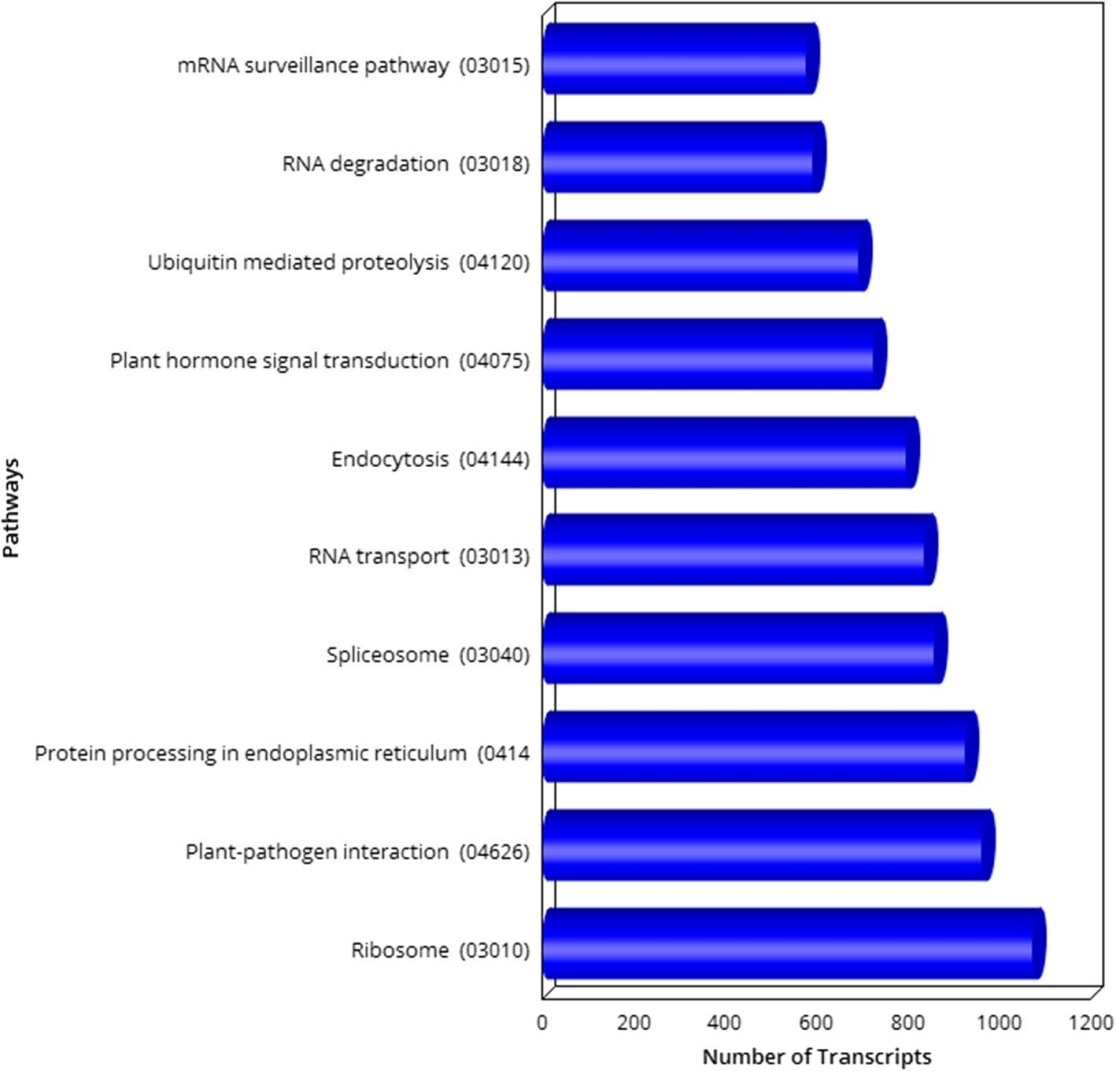
Top 10 highly represented pathway distribution

KAAS Server was used for pathway analysis. For identifying pathways, plant model organisms were considered as reference. Total of 14,063 transcripts were annotated against KAAS out of which “Ribosome” pathway was found to be most abundant followed by “Plant -Pathogen interaction”, “Protein processing in endoplasmic reticulum” etc. (Fig. 4).

**Fig. 5:**
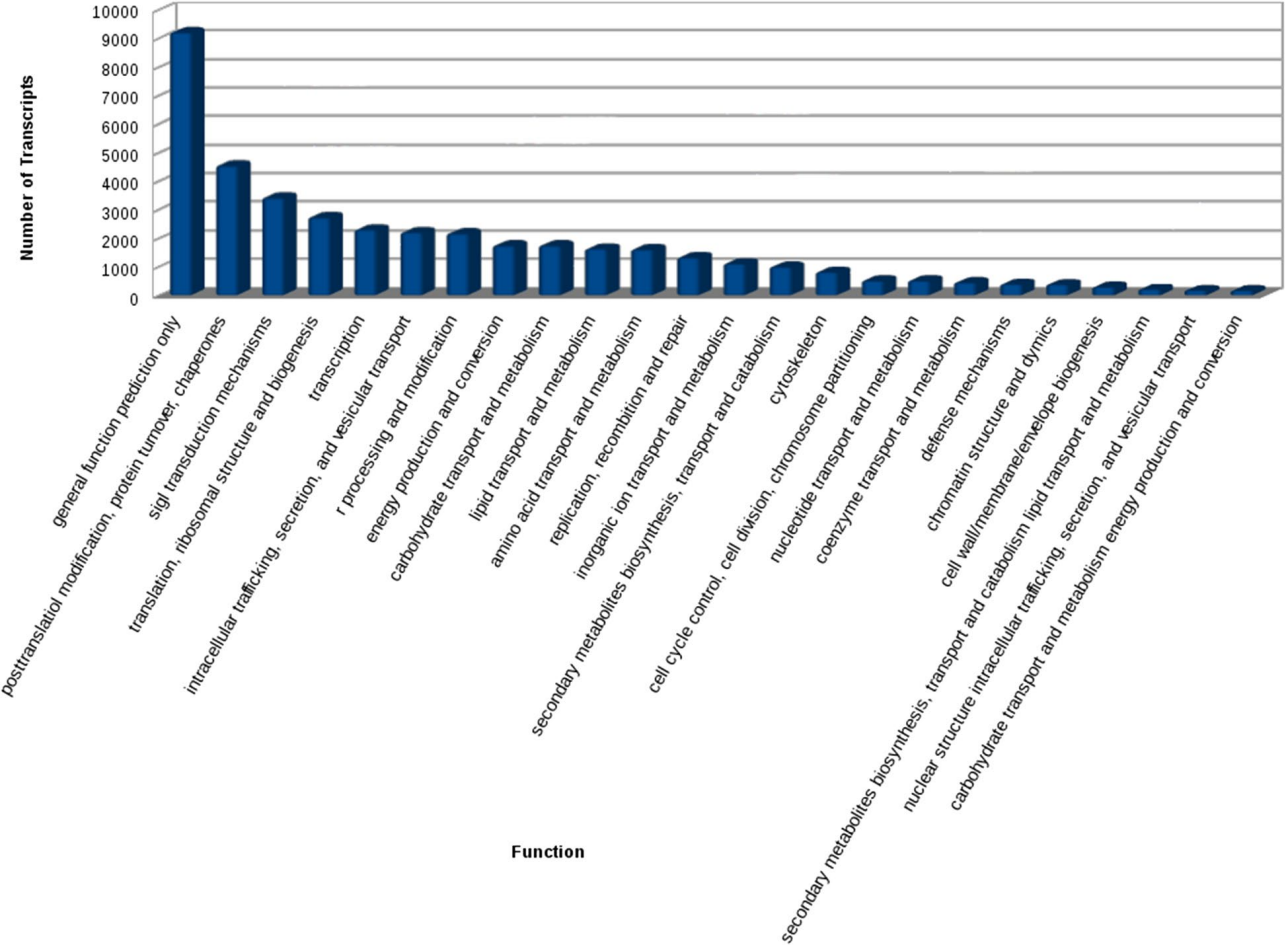
Top 25 KOG functions

**Fig. 6:**
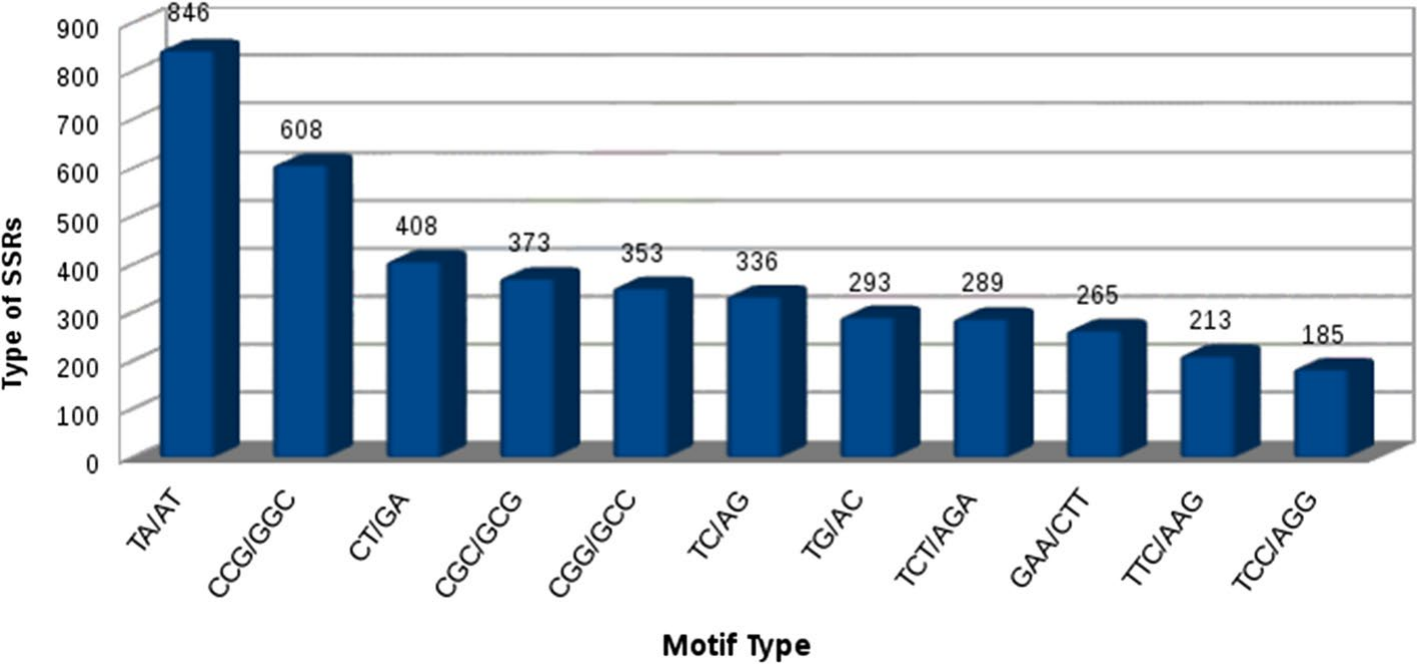
SSR motif type distribution. The most abundant SSR motif was TA/AT

Total of 58% transcripts were annotated against KOG database. Top 25 KOG functions were plotted (Fig. 5). The homology search was carried out using NCBI BLAST 2.2.29. MISA perl script was used for the identification of Simple Sequence Repeats (SSRs) in the transcripts. It was found that 27.02% of the transcripts have SSRs. Total of 33,890 SSRs were predicted in 125,397 transcripts. However, 5732 transcripts had more than one SSR. Most of the SSRs were mono-nucleotide and tri-nucleotide repeats (Additional file 7). TA/AT motifs were observed to be the highest distributed SSR motif type reaching a total of 846, followed by CCG/GGC reaching a total of 608 (Fig. 6).

The gross RPKM for all the identified enzymes in each pathway was analyzed (Additional file 8). The most expressed biosynthetic pathway was thus found to be the phenylpropanoid pathway, followed by methylglyoxal degradation pathway. Terpenoid pathway was the third most expressed secondary metabolite pathway, followed by the flavonoid pathway.

Based on the highest RPKM observed for PAL and C4H, the corresponding reactions catalyzed by these enzymes are assumed to be the most prominent in the phenylpropanoid metabolic pathway in black pepper i.e., the trans-cinnamate biosynthesis and trans-4-coumarate biosynthesis. These two processes result in the biosynthesis of trans-cinnamate from L-phenylalanine and trans-4-coumarate from trans-cinnamate respectively [24, 25]. The most expressed pathway being the phenylpropanoid pathway, the enzyme participants of the pathway were identified from the transcriptome data, which included phenylalanine ammonia lyase (PAL), cinnamate-4-hydroxylase (C4H), 4-coumarate CoA Ligase (4CL), cinnamoylreductase (CCR), hydroxyl cinnamoyltransferase (HCT), cinnamyl alcohol dehydrogenase (CAD), Caffeic acid 3-O-methyltransferase (COMT) and p-coumaroyl ester 3'-hydroxylase (C3'H) [26]. Most of the enzymes identified belonged to the flavonone and monolignol biosynthetic branches in the phenylpropanoid pathway [27], and were selected for further study.

### Real-time validation studies

The expression of the phenylpropanoid biosynthetic genes identified from the transcriptomic data were compared in the different developmental stages of *P. nigrum* berries using qRT-PCR. The details of the RNA samples used are given in Additional file 9.

**Fig. 7:**
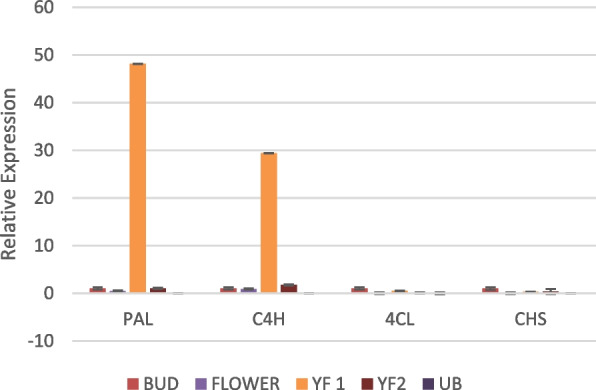
Real-time expression analysis of PAL, C4H, 4CL and CHS. The expression level in unripe berry is too low to be seen near that of YF2 in most cases. High expression was observed in YF1 for PAL and C4H

**Fig. 8:**
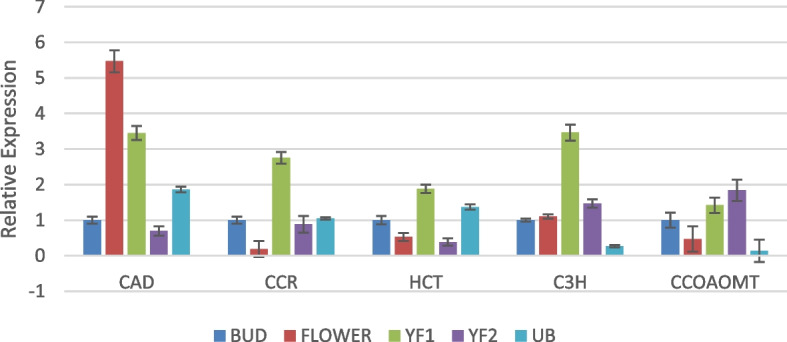
Real-time expression analysis of cinnamyl alcohol dehydrogenase (CAD), cinnamyl CoA reductase (CCR) shikimate O-hydroxycinnamoyltransferase (HCT), p-coumarate 3-hydroxylase (C3H) and caffeoyl-CoA O-methyltransferase (CCOAOMT)

**Fig. 9:**
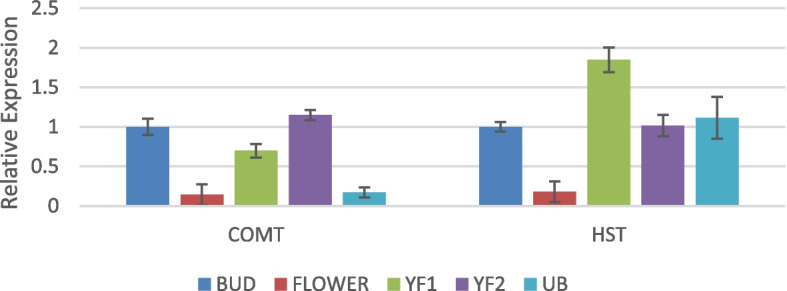
Real-time expression analysis of caffeic acid 3-O-methyltransferase (COMT) and hydroxycinnamoyl-Coenzyme A shikimate/quinate hydroxycinnamoyltransferase (HST)

The general phenylpropanoid pathway consist of the first four enzymes PAL, C4H, 4CL and CHS, and so it was significant to observe that the levels of these enzymes dropped in unripe berries (Fig. 7). The first enzyme of the phenylpropanoid pathway, PAL showed highest expression in the young fruits stage 1 as compared to bud, flower, young fruit stage 2 and unripe berry. PAL catalyses the deamination of phenylalanine to trans-cinnamate. Thus it connects the flux of carbon from shikimate pathway to the phenylpropanoid pathway [28]. C4H also showed similar expression level. However, 4CL and CHS showed abundance in bud stage. The succeeding genes of the pathway, such as CAD, CCR, HCT, p-coumarate 3-hydroxylase (C3H), Caffeoyl-CoA O-methyltransferase (CCOAOMT), Caffeic acid 3-O-methyltransferase (COMT) and Hydroxycinnamoyl-Coenzyme A shikimate/quinatehydroxycinnamoyltransferase (HST) that are involved in secondary metabolite production were not high in the unripe berries in comparison with other developmental stages of the fruit (Figs. 8 and 9). CAD was observed to be very high in the flowers. CCR, HCT, C3H and HST also showed abundance in young fruit stage 1. COMT and CCOAOMT were high in young fruit stage 2.

### Expression analysis of genes encoding transcription factors

Based on the observation of gene expression, certain genes encoding transcription factors were also picked up from the transcriptome data that were associated with the phenylpropanoid pathway. The selected transcription factors included MYB1R1, R2R3MYB, WD40, WRKY2, WRKY33, and LIM. The repressing activity of MYB1R1 on PAL, C4H, HCT, C3H, COMT, CCOAOMT as well that of R2R3MYB on COMT and C4H was reported. WD40, WRKY2 and WRKY33 are known to be activators of CHS gene, promoter of C4H and defense-related genes respectively. LIM is a known transcriptional activator of PAL, 4CL and CAD [29–32]. The quantitative real-time expression analysis was performed for the genes encoding transcription factors. The expression level was analysed in comparison to bud, in all cases.

**Fig. 10:**
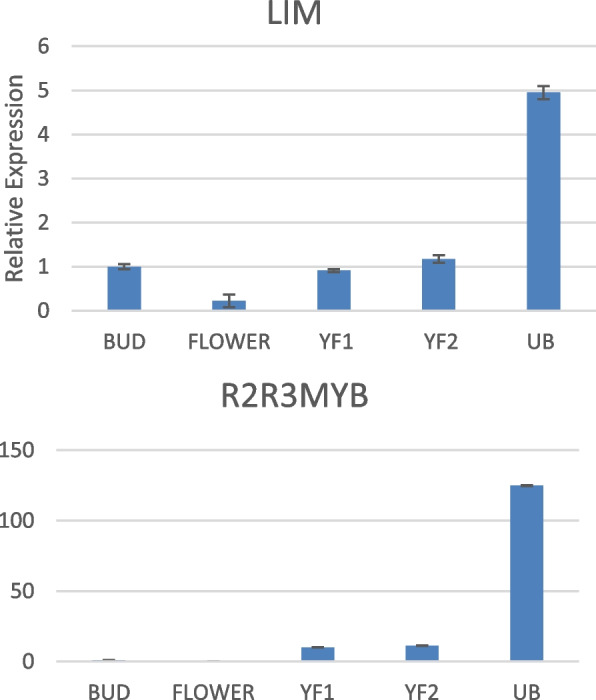
Expression analysis of the transcription factors LIM and R2R3MYB

**Fig. 11:**
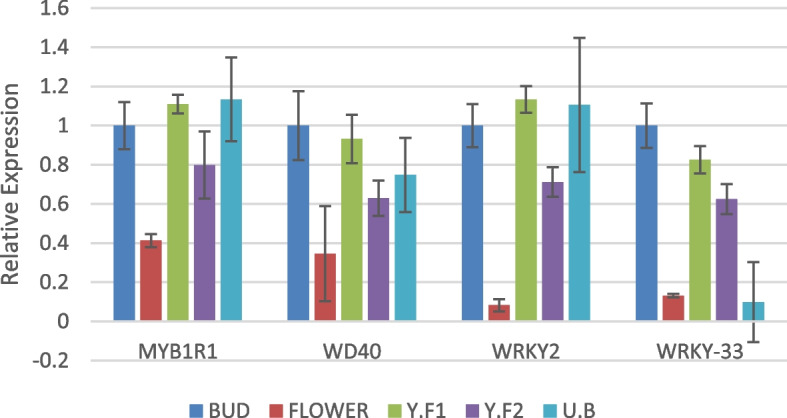
Expression analysis of the transcription factors MYB1R1, WD40, WRKY2 and WRKY33

When we looked into the expression of transcription factors, we observed that R2R3MYB, LIM transcription factor and MYB1R1 were highly abundant in unripe berries. WD40 and WRKY33 showed high expression in bud, whereas WRKY2 in young fruit stage 1 (Figs. 10 and 11). The trend of expression of the phenylpropanoid pathway genes may be the reflection of the activity of the transcription factors. But in some cases, it was different from what was expected. For instance, LIM, which enhances the transcriptional activation of the genes PAL, 4CL and CAD, is high in UB compared to YF1. However, an increased expression of these genes was observed in YF1.

**Fig. 12:**
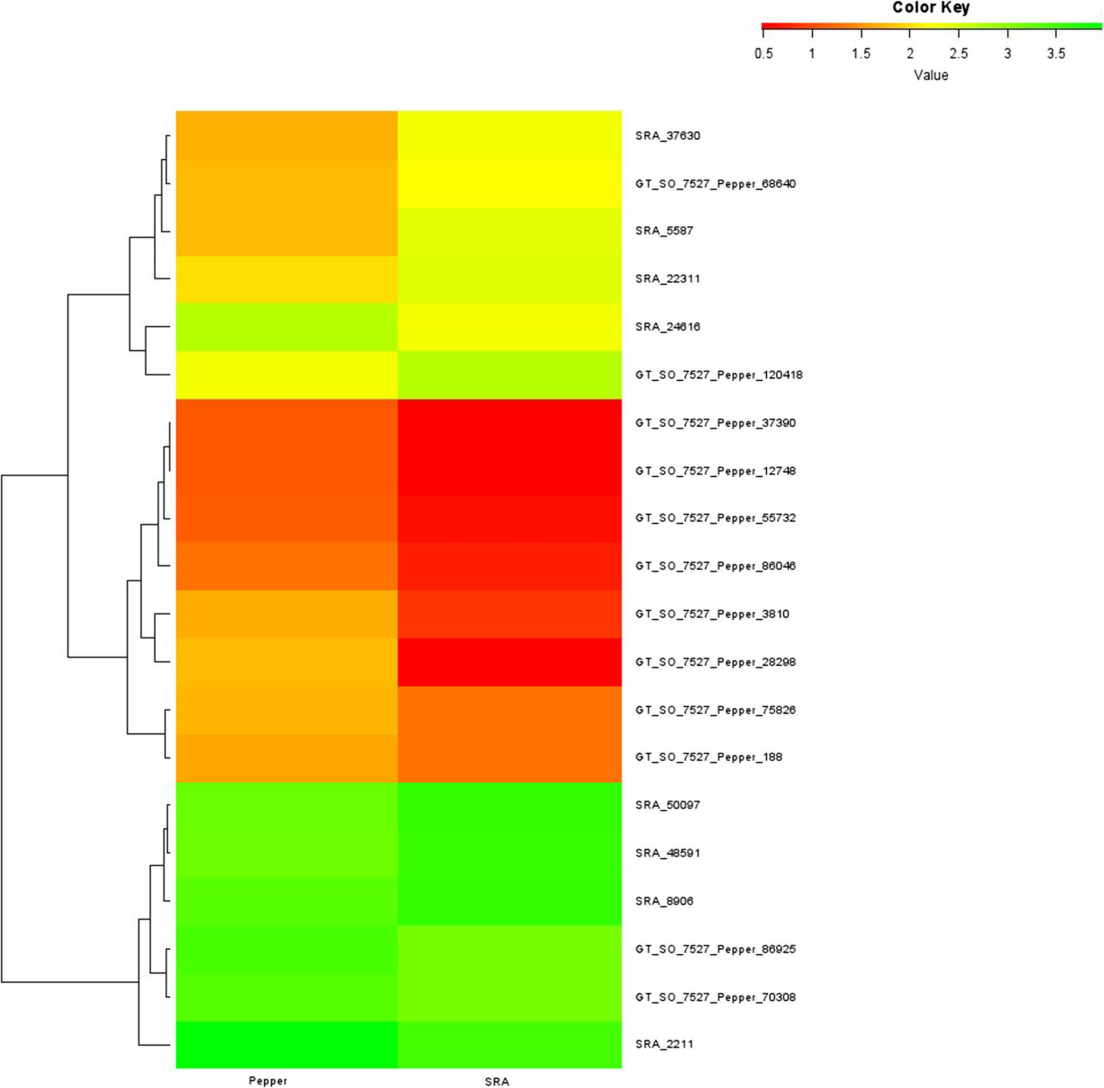
Heat map-based comparison with previous data. Comparison of the unripe *P. nigrum* berry transcriptome RPKM data (‘Pepper’) with the previous RPKM data obtained by [24] using pooled sample of berries of different stages of *P. nigrum* (‘SRA’)

There are reports of previous transcriptome attempts in black pepper with variation in sample and other conditions [10, 23, 33, 34]. We compared the RPKM of selected genes from our data with that of [24] retrieved from NCBI Sequence Read Archive (SRA) (http://www.ncbi.nlm.nih.gov/Traces/sra/; accession number SRS856941) in which they used a mixture of RNA from different stages of fruit (1–10 months post anthesis). None of the selected genes showed significant variation in RPKM (more than 2 fold) between the sample of the current study and that of the previous one (Fig. 12).

## Discussion

The phenylpropanoid pathway is a complex network that has a set of important compounds undergoing modifications with the help of many enzymes. This results in a huge set of secondary metabolites spread over several branches [35, 36]. An enzyme can be responsible for synthesizing more than one metabolite that may be related to each other structurally [37].The complete information on the set of enzymes active at a particular stage can be uncovered using NGS technology. NGS technology helps in the identification of potential candidates that can be targeted to enhance the quality of fruits in *P. nigrum* [38].

When the fruits begin to develop, it is light green in colour and on maturation, colour darkens and the berry becomes harder [39]. The commercially available black pepper is prepared by a procedure involving hot water soaking and drying of the mature green unripe fruits. The high level of phenylpropanoids provides resistance to biotic and abiotic stress besides imparting aroma and spiciness [24]. Transcriptome data help to reveal the important enzymes and regulatory factors connected with the phenylpropanoid pathway in *P. nigrum* berries [40]. Several genes in a biosynthetic pathway can be regulated at the same time by over-expressing transcription factors through successful methods [41]. Transcription factors are reported to be promising candidates for crop improvement using transgenics [42].

A wide array of genes implicated in secondary metabolism in *P. nigrum* berries were identified from the gene ontology annotation. PAL is the first enzyme in phenylpropanoid pathway [43]. CCoAOMT is a participant of monolignol biosynthetic process [44]. COMT, HCT, CCR and CAD are the key enzymes associated with the biosynthesis of lignin [18, 44–46]. The precursors of lignin are produced by the involvement of the enzymes C4H, 4CL and CAD [47]. C4H is necessary for the conversion of trans-cinnamic acid into p-coumaric acid. C4H also supervises the carbon flux for phytoalexins formed during stress conditions [48]. It is a key enzyme linked to the synthesis of many phytochemicals since p-coumarate serves as a common intermediate in several pathways [49]. 4CL, the last enzyme of phenylpropanoid metabolism, converts p-coumarate into its precursor ester to lead to the formation of lignins and flavonoids [35].

The monomers of lignin are called monolignols, a set of significant precursors formed through the phenylpropanoid pathway related to development and defense [35]. The branching point in the phenylpropanoid pathway into flavonoid pathway and monolignol biosynthetic pathway starts at the metabolite p-coumaroyl CoA. This compound can be a substrate for CHS resulting in the beginning of flavonoid pathway or for HCT, leading to monolignol synthesis [50]. HCT gene converts p-coumaroyl CoA into caffeoyl CoA and feruloyl CoA [51]. Negative regulation of one or a set of genes in the lignin biosynthetic branch can cause variation in the metabolic flux due to the lowered carbon flow towards the lignin pathway. Thus it depends on whether the key compound p-coumaroyl CoA serves as a substrate to HCT or CHS to determine the direction of the metabolic flux towards the monolignol or flavonoid branches of the phenylpropanoid pathway [50]. Any change in the regulation of a particular branch of the phenylpropanoid pathway is expected to immensely affect the other branches and their corresponding substrates [36]. Using metabolic engineering, it is possible to accumulate valuable metabolites by the extension of biosynthetic branches or addition of novel branches [52]. Better accumulation of desirable metabolites in *P. nigrum* fruit for industrial and pharmaceutical purposes can be obtained by hindering the competitive branches of biosynthesis [53].

Gene expression analysis helps to confirm the results obtained from RNA-Seq data [38]. The expression of selected genes was analysed in different developmental stages using qRT-PCR experiment. The expression pattern varied considerably in the different organs of *P. nigrum* i.e., bud, flower, young fruits stage 1 and 2 as well as unripe mature berries. In comparison with the bud stage, the expression of genes belonging to the phenylpropanoid pathway was lower in unripe berries in the majority of cases.

It is evident that the expression level of CHS is lower than HCT in the different developmental stages. This is in accordance with the trend in the transcriptomic data which showed the higher expression of monolignol biosynthetic genes in comparison with flavonoid pathway genes. It is to be noted that the most prominent pathway was identified as the phenylpropanoid pathway. Flavonoid pathway was only the 4^th^ among the most active pathways in *P. nigrum*.

The qRT-PCR results showed that it was feasible to select a particular developmental stage for the metabolic engineering strategy to accumulate a specific bioactive compound. Thus the conventionally used developmental stages of fruits are not necessarily the only stages that have bioactivity. The other stages can also be considered for the isolation of specific bioactive compounds based on further confirmative studies in future.

Contrasting trends observed in gene expression may be a reflection of differential control of transcription [54]. To further look into this, we explored the putative transcription factors associated with the phenylpropanoid pathway in *P.nigrum*. Phenylpropanoid metabolism is reported to be regulated by highly conserved transcription factors like MYB and WD40 [55, 56]. In plants, MYB is the largest family of transcription factors with a MYB domain that is conserved in nature [57]. MYB regulates C4H and caffeic acid 3-O-methyltransferase (COMT) genes [44]. MYB transcription factors show repressing activity to the expression of cinnamate 4-hydroxylase (C4H) gene by directly regulating corepressors that aid in the binding of MYB protein with the promoter of C4H gene [58]. If MYB transcription factor is repressed, it may direct the pathway towards lignin biosynthesis [59].

As per previous reports, the downregulation of CCoAOMT in the MYB-over expressed condition results in low lignin production in tobacco [60]. VvMYB4a and VvMYB4b of *Vitis vinifera* may negatively regulate the synthesis of low molecular weight phenolic compounds by inhibiting certain genes involved in the phenylpropanoid biosynthesis [61]. MYB/bHLH/WD-repeat (MBW) family genes play significant role in the flavonoid pathway in Arabidopsis [44].

The regulation of phenylpropanoid pathway by R2R3-MYB transcription factors was evidently demonstrated [62]. Overexpression of R2R3-MYB transcription factor has adversely affected several genes in the phenylpropanoid pathway that reduced the accumulation of lignin [63]. These transcription factors repress flavonoid and phenylpropanoid pathway genes that help in the production of anthocyanins and hydroxycinnamic acid esters [64]. R2R3-MYB transcription factor functions in the regulation of flavonoid pathway and monolignol pathway [57, 65, 66], whereas it shows repressing activity on the phenylpropanoid enzymes COMT and C4H [30–32].

WD40 (also known as the WD or beta-transducin repeat) is implicated in the accumulation of phenylpropanoids in *Solanum tuberosum* [67]. WRKY transcription factors contain distinctive WRKY domains exclusive to plants and play indispensable role in defense [68]. The expression analysis of genes encoding WRKY transcription factors in response to abiotic stress was studied in different tissues of pearl millet, providing underpinning data for their application in improving crops [69, 70]. LIM helps in increasing lignin production by enhancing the activity of phenylalanine ammonia‐lyase, 4-coumarate-CoA ligase and cinnamyl alcohol dehydrogenase genes of the phenylpropanoid pathway [71]. The comparatively lower expression of the genes observed in the real-time expression analysis may be a reflection of the activity of the transcription factors. However, in addition to transcription factors, post-transcriptional modifications also play an important role in deciding metabolic shifts [54]. Contradicting expression trends of some genes regulated by the transcription factors suggest the involvement of additional regulatory factors which needs further experimental evidence.

There are reports of previous transcriptome attempts in black pepper with variation in sample and other conditions [10, 23, 33, 34]. The comparison of the results obtained in this study needs to be confirmed using the recently published reference genome of black pepper [72]. However, we compared the expression of selected genes from our data with that of [24] with the help of a heat map (Fig. 12). They used pooled sample of different stages of fruit (1 to 10 months after flowering) from *P. nigrum* cv. Reyin 1 whereas we used the mature unripe berries alone from which black pepper is produced commercially. Though there was a general variation in the intensity, a similar trend of expression of genes and transcription factors was observed. Altered source–sink communications are assumed to have resulted in the observed trend of gene expression [54].

## Conclusion

The *de novo* transcriptome profile of unripe *P. nigrum* berries brought out the prominent secondary metabolic pathways, of which the highest expressed genes belonged to the phenylpropanoid pathway. The differential expression of these genes and significant transcription factors regulating the pathway were analyzed in the various developmental stages from bud to the development of the green berries from which black pepper is prepared for commercial applications. The expression of the selected transcripts was compared with previously published transcriptome data of a pooled sample of *P. nigrum* fruits of different developmental stages. The significant information obtained from the study can be beneficial for the commercial production of natural pharmaceuticals as well as for metabolic engineering studies that aim for black pepper berries enriched in a particular secondary metabolite group.

## Supplementary Information

**Additional file 1.**

**Additional file 2.**

**Additional file 3.**

**Additional file 4.**

**Additional file 5.**

**Additional file 6.**

**Additional file 7.**

**Additional file 8.**

**Additional file 9.**

## Data Availability

“The datasets generated and/or analysed during the current study are available in the [NCBI SRANAME] repository, [PER PRJNA873239 and PRJNA872302SISTENT WEB LINK OR ACCESSION NUMBER TO DATASETS]”.
