# Supplementary material for: De novo transcriptome profiling unveils the regulation of phenylpropanoid biosynthesis in unripe Piper nigrum berries
Source: BMC Plant Biol. 2022 Oct 26;22:501. doi: 10.1186/s12870-022-03878-1 (PMC9597958; doi:10.1186/s12870-022-03878-1)
Supplement: Supplementary file 1 — Additional file 1. [file 12870_2022_3878_MOESM1_ESM.docx]

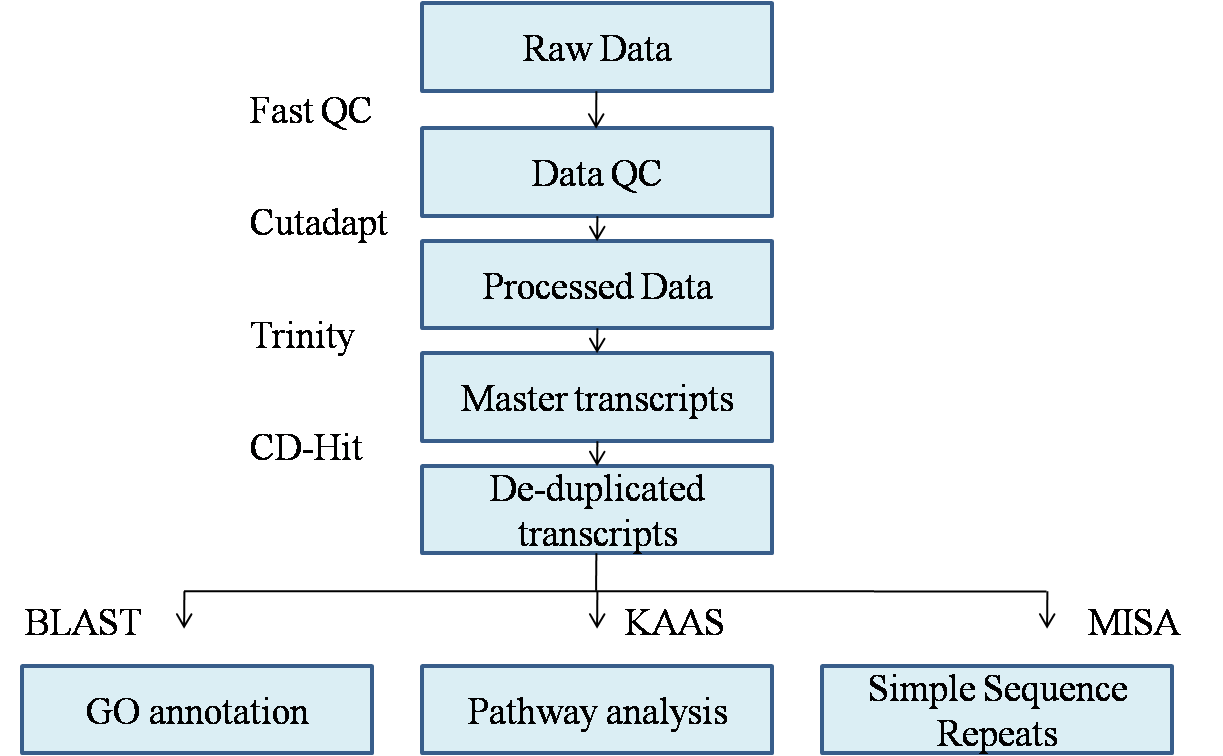


**Workflow illustrating the *de-novo* transcriptome analysis**. The functional annotation included BLAST, KAAS and MISA.
