# Supplementary material for: De novo transcriptome profiling unveils the regulation of phenylpropanoid biosynthesis in unripe Piper nigrum berries
Source: BMC Plant Biol. 2022 Oct 26;22:501. doi: 10.1186/s12870-022-03878-1 (PMC9597958; doi:10.1186/s12870-022-03878-1)
Supplement: Supplementary file 2 — Additional file 2. [file 12870_2022_3878_MOESM2_ESM.docx]

| **Sl. No.** | **Primer ID** | **Primer Sequence (5'-3')** | **Length** |
| --- | --- | --- | --- |
|  | PAL FP | AGGTTCGTTAGGGAGGAGTT | 20 |
|  | PAL RP | GCATTCCAAGAGTGGGTCTATC | 22 |
|  | C4H FP | ACAACTACGGCGACTTCATC | 20 |
|  | C4H RP | GGCTTGGTGCTAGACATCTT | 20 |
|  | CHS FP | CAAGATATGGTGGTGGTGGAG | 20 |
|  | CHS RP | CAGAAGATGAGGTGGGTGATG | 20 |
|  | 4CL FP | CCATCTCCCTCTTCATGCTTAC | 22 |
|  | 4CL RP | TGGGTCTCTGGAAACGAGTA | 20 |
|  | CAD FP | TTTCGATTTGATGGGCCAAAG | 21 |
|  | CAD RP | GCTAGCATTAATGGTCCTGTTG | 22 |
|  | CCR FP | TGCATAAGGCGCGGATTT | 18 |
|  | CCR RP | GCCAGGCTGCGAATCTTT | 18 |
|  | HCT FP | CTTCTTCGACATGGCCGATA | 20 |
|  | HCT RP | GCTACCATCTCTGGACAATCTC | 22 |
|  | C3H FP | GGTCACCTTCTGCACCATTT | 20 |
|  | C3H RP | AGAGGAGTCCGCATGTATGT | 20 |
|  | CCOA-OMT FP | CGAGACTTCGTTCTGGAGTTG | 21 |
|  | CCOA-OMT RP | TTGATGCGACGGCAAAGA | 18 |
|  | MYB1R1 FP | CTTGGGTAGGAGTCCTTGAAAC | 22 |
|  | MYB1R1 RP | GACTCCTTGGACTGAAGAAGAAC | 23 |
|  | R2R3MYB FP | GAACCGACAATGAGGTGAAGA | 21 |
|  | R2R3MYB RP | GGAGAGAATGCAGACGATGG | 20 |
|  | WD40 FP | CTTGATGGCTTGCCTCAGAA | 20 |
|  | WD40 RP | CACTCTTGTTGCTCCCTGTATC | 22 |
|  | WRKY2 FP | CCCAGATGTGAGGTCTTCATAC | 22 |
|  | WRKY2 RP | GCAGGGTCAGGTAATTGGATAG | 22 |
|  | WRKY33 FP | CCTGTTCTCCTCTCCACTTCTA | 22 |
|  | WRKY33 RP | TGATGCCTGGTGGGTATTTG | 20 |
|  | COMT FP | GAGGCTGCTATGCCAACTATT | 21 |
|  | COMT RP | TCCCTTTGGGTCCTCTCTTT | 20 |
|  | HST FP | AGACCTGCTCTGTTCTGGA | 19 |
|  | HST RP | GCAGTCAACGGACGGAATAA | 20 |
|  | C3’H FP | CAAGGAGGTGCTCAAGGAAA | 20 |
|  | C3’H RP | CACCTTCCTCACCTTCACATAG | 22 |
|  | LIM FP | CAGTCTTCTTACAGGCCACAC | 21 |
|  | LIM RP | TGATTGAGCAGTCCAGCAAA | 20 |
|  | 18S FP | AGACGAACAACTGCGAAAGC | 20 |
|  | 18S RP | GCGGAGTCCTAAAAGCAACA | 20 |
|  | 5.8S FP | CGTAGCGAAATGCGATACTTGGTGTG | 26 |
|  | 5.8S RP | CAACTTGCGTTCAAAGACTCGATGGT | 26 |

**Details of the primers.** The sequences and length of forward primer (FP) and reverse primer (RP) for each of the genes and transcription factors used for the study.
