# Supplementary material for: De novo transcriptome profiling unveils the regulation of phenylpropanoid biosynthesis in unripe Piper nigrum berries
Source: BMC Plant Biol. 2022 Oct 26;22:501. doi: 10.1186/s12870-022-03878-1 (PMC9597958; doi:10.1186/s12870-022-03878-1)
Supplement: Supplementary file 3 — Additional file 3. [file 12870_2022_3878_MOESM3_ESM.docx]

| **Concentration** | **260/280 ratio** | **260/230** | **RNA integrity number (RIN)** |
| --- | --- | --- | --- |
| 513.6 ng/µl | 2.16 | 2.29 | 8.6 |

**Concentration and quality of RNA sample.** The details of the high quality RNA used for NGS.
