# Supplementary figures and images for: De novo transcriptome profiling unveils the regulation of phenylpropanoid biosynthesis in unripe Piper nigrum berries
Source: BMC Plant Biol. 2022 Oct 26;22:501. doi: 10.1186/s12870-022-03878-1 (PMC9597958; doi:10.1186/s12870-022-03878-1)

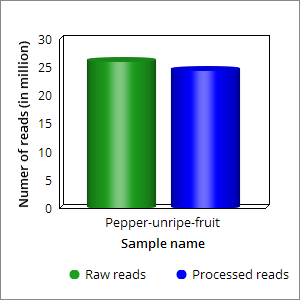


**Read distribution**. Number of raw reads and processed reads in million

Supplement: Supplementary file 4 — Additional file 4. [file 12870_2022_3878_MOESM4_ESM.docx]

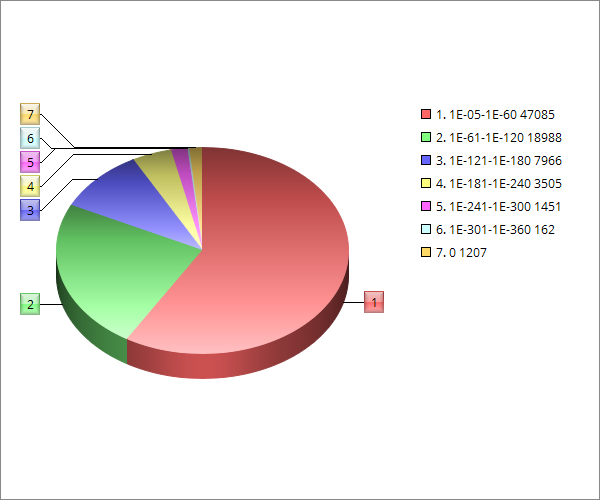


**E-value distribution plot**. 47085 transcripts with high similarity (E-value <1e-60) were observed.

Supplement: Supplementary file 5 — Additional file 5. [file 12870_2022_3878_MOESM5_ESM.docx]

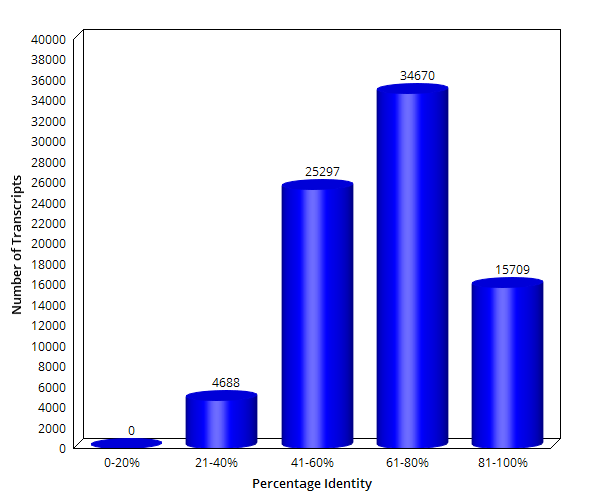


**Percentage identity plot.** 15, 709 sequences had a similarity higher than 80%

Supplement: Supplementary file 6 — Additional file 6. [file 12870_2022_3878_MOESM6_ESM.docx]
