# Supplementary material for: De novo transcriptome profiling unveils the regulation of phenylpropanoid biosynthesis in unripe Piper nigrum berries
Source: BMC Plant Biol. 2022 Oct 26;22:501. doi: 10.1186/s12870-022-03878-1 (PMC9597958; doi:10.1186/s12870-022-03878-1)
Supplement: Supplementary file 7 — Additional file 7. [file 12870_2022_3878_MOESM7_ESM.docx]

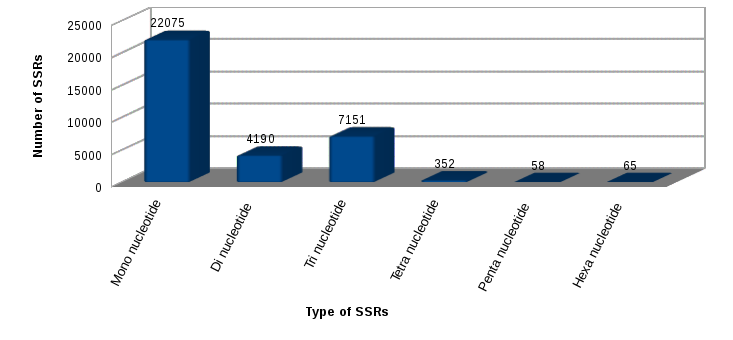


**SSR distribution in different classes.** The majority of SSRs were mono-nucleotide and tri-nucleotide repeats.
