# Supplementary material for: De novo transcriptome profiling unveils the regulation of phenylpropanoid biosynthesis in unripe Piper nigrum berries
Source: BMC Plant Biol. 2022 Oct 26;22:501. doi: 10.1186/s12870-022-03878-1 (PMC9597958; doi:10.1186/s12870-022-03878-1)
Supplement: Supplementary file 9 — Additional file 9. [file 12870_2022_3878_MOESM9_ESM.docx]

| **Sample** | **Concentration (ng/µl)** | **260/280 ratio** | **260/230** |
| --- | --- | --- | --- |
| Bud | 228.30 | 2.11 | 2.19 |
| Flower | 238.06 | 2.12 | 2.19 |
| Y.F1 | 145.09 | 2.05 | 1.94 |
| Y.F2 | 238.00 | 2.04 | 1.85 |
| UB | 341.90 | 2.11 | 2.17 |

**Concentration and quality of RNA samples.** High quality RNA was used for qRT-PCR studies
